# Risk of venous thromboembolism and benefits of prophylaxis use in hospitalized medically ill US patients up to 180 days post-hospital discharge

**DOI:** 10.1186/1477-9560-9-15

**Published:** 2011-10-13

**Authors:** Li Wang, Nishan Sengupta, Onur Baser

**Affiliations:** 1 STATinMED Research, Ann Arbor, MI, USA; 2 Janssen Global Services, Raritan, NJ, USA; 3 STATinMED Research and The University of Michigan, Ann Arbor, MI, USA

## Abstract

**Background:**

To assess the incidence of venous thromboembolism (VTE) and bleeding events with or without thromboprophylaxis and the associated costs in a cohort of medically ill patients in both in-hospital and outpatient settings.

**Methods:**

A large hospital drug database and linked outpatient files were used to identify patients eligible for this analysis, based on demographic and clinical characteristics.

**Results:**

Among 11,135 patients identified, 1592 (14.30%) were admitted with chronic heart failure, 1684 (15.12%) with thromboembolic stroke, 3834 (34.43%) with severe lung disease, 1658 (14.89%) with acute infection, and 2367 (21.26%) with cancer. Of the 11,135 patients, 5932 received anticoagulant therapy at some point during their hospitalization and until 30 days after discharge. VTE events occurred in 1.30% of patients who received anticoagulant prophylaxis versus 2.99% of patients who did not. Risk-adjusted total healthcare costs for patients with a VTE or major or minor bleeding event were significantly higher than for those without events (VTE: $52,157 ± 24,389 vs $24,164 ± 11,418; major bleeding: $33,656 ± 18,196 vs $24,765 ± 11,974; minor bleeding: $33,690 ± 14,398 vs $23,610 ± 11,873). In a multivariate analysis, appropriate anticoagulant prophylaxis use was significantly associated with a reduced risk of clinical VTE, compared with no anticoagulant use (hazard ratio: 0.37). Patients admitted with thromboembolic stroke were less likely to have a VTE than patients admitted with cancer (hazard ratio: 0.42).

**Conclusions:**

In this analysis, VTE and major bleeding event rates were lower for patients who received prophylaxis compared with those who did not. Prophylaxis use was associated with lower healthcare costs.

## Background

Venous thromboembolism (VTE), including deep vein thrombosis (DVT) and pulmonary embolism (PE), is an important cause of disability and death. Many patients require hospitalization, and the annual death rate from VTE is estimated to be as high as 300,000 persons per year [1]. Various medical diseases confer a high risk of VTE, including malignant neoplasms, with and without chemotherapy; prior superficial vein thrombosis; and neurological diseases with extremity paresis [2]. Among hospitalized non-surgical patients, VTE risk is especially high in those who are critically ill. Moderate to high risk of VTE has been documented in patients with acute myocardial infarction (MI; 24%) and in the paretic or paralyzed lower extremity of ischemic stroke patients (55%) [3].

The American College of Chest Physicians (ACCP) guidelines on antithrombotic therapy recommend the use of pharmacologic thromboprophylaxis in hospitalized medical patients at significant risk of VTE [3,4]. However, despite these guidelines, VTE prophylaxis is underutilized for medical non-surgical patients compared with surgical patients [4-6].

While results of randomized trials or prospective observational studies could determine the benefits of VTE prophylaxis in hospitalized medically ill patients, this approach would be both expensive and time consuming. Moreover, in areas such as VTE prophylaxis, real-world practices often significantly diverge from clinical trial methodologies. The present analysis attempts to address this problem. We have used an electronic hospital drug database with linked outpatient data to specifically investigate the use of thromboprophylaxis and rates of outcomes events such as VTE, major bleeding, minor bleeding, and costs among medically ill patients, both during hospitalization and post-discharge.

## Methods

### Data Sources and Study Population

This analysis used a subset of the *MarketScan*^® ^databases and linked outpatient files from the *Market Scan *Commercial and Medicare Supplemental databases from *Thomson Reuters*. The *Market Scan *Commercial and *Market Scan *Medicare Supplemental databases [7] include submitted claims (UB02 format) linked to detailed service-level hospital bills for the same admissions. By 2007, data for nearly 22,189 patient-level hospital records had been successfully linked to longitudinal claims histories in the *Market Scan *Commercial and *Market Scan *Medicare Supplemental databases. The linkage was conducted for 172 hospitals identifiable in both the hospital and claims databases. The data include full census annual admissions for each contributing hospital. Claims data are fully adjudicated and paid claims from covered medical and pharmacy services are from geographically dispersed private and public health plans [8].

For our analysis, the following patient-level identifiers were used to match the claims and inpatient databases: admission date, discharge date, age, sex, and principal diagnosis; cases that were uniquely characterized by these variables were identified. The paid claims were linked with a record that matched the same combination of these variables. This match was considered to be the same patient, given that each combination of matching variables was unique within the hospital census [9].

For this analysis, data from commercial and Medicare patients, between January 1, 2005, and December 31, 2007, with a first principal medical diagnosis that fell into the acute medical illness category were considered for inclusion. Categories included: (1) chronic heart failure, (2) thromboembolic stroke, (3) severe lung disease, (4) acute infection, and (5) cancer. (See Appendix for ICD-9-CM codes used to identify medical conditions.) For inclusion, all patients must have had continuous enrollment in their health plans for ≥180 days prior to and 180 days following the date of admission, which was defined as the index date.

Demographic (age, gender, geographic location) and clinical characteristics were derived from the claims database. The Deyo adaptation of the Charlson Comorbidity Index [10-12] was used to calculate relative baseline comorbidities for each patient. Presence of hypertension, renal disease, and cancer (excluding myelodysplastic syndrome) during the baseline period were also identified, and baseline characteristics were compared among the patient cohorts. Descriptive statistics were calculated as means ± standard deviation (SD) and percentages. Differences between the cohorts were analyzed using the *t*-test, Mann-Whitney *U *test, and chi-square test.

The timing of use of any guideline-recommended anticoagulant--low molecular weight heparin (LMWH), warfarin, unfractionated heparin (UFH), or fondaparinux--was measured from the index date until 180 days after hospital discharge. In order to determine whether prophylaxis use was appropriate according to published guidelines [4], patients had to have received any anticoagulant after the index date and within 30 days of hospital discharge and before the date of their first clinical VTE.

Patients who developed clinical VTE were identified on the basis of: (1) a primary or secondary diagnosis for DVT (ICD-9-CM 451.1x-451.81, 451.83-451.9x or 452.xx or 453.2-453.9x) or PE (ICD-9-CM: 415.1x) on the medical claim between the index date and 180 days after hospital discharge, and (2) receipt of an anticoagulant drug within 15 days of the date of this claim.

All outcomes were assessed 180 days from the index hospitalization. In addition to the incidence of VTE events, the incidence of major and minor bleeding was also assessed, using the appropriate ICD-9-CM codes. The median time to all outcome events was estimated using the Cox proportional hazard regression model. Costs for 180 days post-discharge were calculated to compare patients in the no-prophylaxis cohort with patients in the any-anticoagulant prophylaxis cohort.

Logistic regression fits models of the rate of event were used to estimate risk-adjusted event rates within 180 days of discharge. A generalized linear model with a log-link function and gamma distribution was used to assess the independent effects of covariates on total 180-day follow-up costs. Statistical analyses were performed using SAS^® ^v9.2 and STATA^® ^v10 software.

## Results

A total of 11,135 patients from the linked database met the inclusion criteria for medical patients at risk of VTE. The most-common medical diagnosis was severe lung disease (34.43%), followed by cancer (21.26%), thromboembolic stroke (15.12%), acute infection (14.89%), and chronic heart disease (14.30%). The average age of patients was 66 years; 47.8% were men and 52.2% women. Most patients lived in the southern part of the United States. During the 6-month baseline period, the most commonly identified risk factors for VTE were hypertension (39.41%) and diabetes (23.73%). VTE was diagnosed in 2.56% of patients. The total outpatient cost at baseline was $17,815 per patient (Table 1). Of the eligible patients, only 5932 (53.2%) received the appropriate anticoagulant VTE prophylaxis. The rate ranged from 69.3% in chronic heart failure to 46.3% in cancer patients (Table 2). In patients with no appropriate anticoagulant prophylaxis, the rate of clinical VTE over 180 days was 3.65%; however, clinical VTE rates were much lower by 180 days (1.79%) in patients with appropriate anticoagulant prophylaxis (Table 3).

**Table 1:** Demographic and clinical characteristics for medically ill patients*

	Chronic heart failure (N = 1592)		Thromboembolic stroke (N = 1684)		Severe lung disease (N = 3834)		Acute infection (N = 1658)		Cancer (N = 2367)		Total (n = 11,135)	
	Mean, n	SD, %	Mean, n	SD, %	Mean, n	SD, %	Mean, n	SD, %	Mean, n	SD, %	Mean, N	SD, %
Age, yr (mean)	71.13	12.97	70.67	12.06	64.04	20.20	63.59	20.01	63.48	13.20	65.87	17.14
Median	74		73		69		67		64		69	
Max	99		97		101		100		99		101	
Min	23		20		1		1		2		1	
0-64	471	29.59%	523	31.06%	1596	41.63%	762	45.96%	1256	53.06%	4608	41.38%
65-74	378	23.74%	416	24.70%	880	22.95%	333	20.08%	604	25.52%	2611	23.45%
75-84	528	33.17%	562	33.37%	1034	26.97%	390	23.52%	424	17.91%	2938	26.39%
85-94	194	12.19%	174	10.33%	311	8.11%	160	9.65%	80	3.38%	919	8.25%
95+	21	1.32%	9	0.53%	13	0.34%	13	0.78%	3	0.13%	59	0.53%
Gender (male)	892	56.03%	873	51.84%	1732	45.17%	738	44.51%	1092	46.13%	5327	47.84%
**Region**												
Northeast	18	1.13%	13	0.77%	74	1.93%	26	1.57%	38	1.61%	169	1.52%
North Central	375	23.56%	358	21.26%	1035	27.00%	401	24.19%	380	16.05%	2549	22.89%
South	1184	74.37%	1291	76.66%	2677	69.82%	1214	73.22%	1920	81.12%	8286	74.41%
West	15	0.94%	22	1.31%	48	1.25%	17	1.03%	29	1.23%	131	1.18%
**Comorbid Conditions (baseline)**												
Elixhauser Comorbidity Index ≥2%	213	13.38%	113	6.71%	343	8.95%	164	9.89%	355	15.00%	1188	10.67%
Charlson Comorbidity Index	1.99	1.85	1.40	1.49	1.46	1.71	1.46	1.92	2.81	2.52	1.81	2.01
Congestive heart failure	792	49.75%	122	7.24%	422	11.01%	183	11.04%	76	3.21%	1595	14.32%
Peripheral arterial disease	105	6.60%	149	8.85%	188	4.90%	91	5.49%	63	2.66%	596	5.35%
Acute coronary syndrome	140	8.79%	50	2.97%	101	2.63%	41	2.47%	37	1.56%	369	3.31%
Hyperthyroidism	12	0.75%	6	0.36%	19	0.50%	7	0.42%	23	0.97%	67	0.60%
Obesity	39	2.45%	18	1.07%	67	1.75%	53	3.20%	33	1.39%	210	1.89%
Diabetes	583	36.62%	422	25.06%	791	20.63%	459	27.68%	387	16.35%	2642	23.73%
Hypertension	747	46.92%	757	44.95%	1273	33.20%	654	39.45%	957	40.43%	4388	39.41%
Ischemic stroke	122	7.66%	680	40.38%	212	5.53%	90	5.43%	89	3.76%	1193	10.71%
Hemorrhagic stroke	4	0.25%	11	0.65%	7	0.18%	6	0.36%	4	0.17%	32	0.29%
Non-CNS systemic embolism	17	1.07%	8	0.48%	50	1.30%	41	2.47%	28	1.18%	144	1.29%
Transient ischemic attack	29	1.82%	170	10.10%	51	1.33%	23	1.39%	21	0.89%	294	2.64%
Catheter ablation	530	33.29%	233	13.84%	437	11.40%	226	13.63%	177	7.48%	1603	14.40%
Dyspepsia	7	0.44%	5	0.30%	17	0.44%	9	0.54%	19	0.80%	57	0.51%
**Healthcare Cost (baseline), $USD**												
Outpatient ED	$205	$531	$179	$546	$205	$650	$263	$790	$198	$1,780	$208	$999
Outpatient office visit	$478	$427	$396	$351	$421	$524	$442	$508	$707	$618	$489	$522
Outpatient pharmacy	$2,019	$2,021	$1,639	$1,909	$2,244	$2,851	$2,151	$2,770	$1,506	$2,610	$1,950	$2,572
Outpatient other	$5,745	$16,318	$4,248	$11,027	$5,302	$15,744	$7,493	$20,180	$11,037	$18,891	$6,752	$16,853
Total outpatient	$8,447	$16,913	$6,462	$11,686	$8,172	$16,607	$10,350	$21,322	$13,448	$20,312	$9,398	$17,815
Baseline VTE	49	3.08%	21	1.25%	85	2.22%	73	4.40%	57	2.41%	285	2.56%
**Admission Diagnosis**												
Chronic heart disease	1592	100.00%									1592	14.30%
Thromboembolic stroke			1684	100.00%							1684	15.12%
Severe lung disease					3834	100.00%					3834	34.43%
Acute infection							1658	100.00%			1658	14.89%
Cancer									2367	100.00%	2367	21.26%

CNS, central nervous system; ED, emergency department; SD, standard deviation; USD, US dollars; VTE, venous thromboembolism.

*6 months prior to admission is pre-period.

**Table 2:** Anticoagulant prophylaxis use among medically ill patients*

	Unadjusted percentage			Risk-adjusted percentage		
Outcome	Appropriate prophylaxis, % (n = 5932)	No prophylaxis, % (n = 5203)	*P*-value	Apppropriate prophylaxis, % (n = 5932)	No prophylaxis, % (n = 5203)	*P*-value
**VTE**	1.79	3.65	<0.0001	1.30	2.99	<0.0001
**Major bleeding**	2.06	1.65	0.1164	1.60	1.54	0.8063
**Minor bleeding**	13.79	12.44	0.0349	13.10	12.25	0.1829
**Readmission**	18.83	12.99	<0.0001	18.34	13.01	<0.0001

VTE, venous thromboembolism.

*All events occurred ≤180 days after discharge.

**Table 3:** Thromboprophylaxis rates in medically ill patients

Medically ill patient subgroup	Patients, N	Patients with appropriate prophylaxis	
		N	%
**Chronic heart failure**	1592	1103	69.3
**Thromboembolic stroke**	1684	1014	60.2
**Severe lung disease**	3834	1947	50.8
**Acute infection**	1658	772	46.6
**Cancer**	2367	1096	46.3

A clear trend for occurrence of all-cause readmission was observed in 12.99% of patients with no appropriate prophylaxis and 18.83% of those with appropriate prophylaxis. The pattern of greater rehospitalization also existed among patients in the any-anticoagulant prophylaxis cohort.

Table 2 presents the risk-adjusted outcome results. The cumulative rate of clinical VTE at 180 days was more than two times higher for patients who did not use appropriate anticoagulant prophylaxis compared with those who did (2.99% vs 1.30%, *p *< 0.0001). Observed rates of major and minor bleeding were similar in the two cohorts.

Table 4 shows that 180-day follow-up costs were significantly associated with outcome events. Of 11,135 patients who had an acute medical illness diagnosed, 296 (2.66%) had clinical VTE, 208 (1.87%) had major bleeding, and 1465 (13.16%) had minor bleeding by 180 days after index hospital discharge. For patients with outcome events, risk-adjusted total healthcare costs were significantly greater than for those without events (VTE: $52,157 ± 24,389 vs $24,164 ± 11,418, *p *< 0.0001; major bleeding: $33,656 ± 18,196 vs $24,765 ± 11,974, *p *< 0.0001; minor bleeding: $33,690 ± 14,398 vs $23,610 ± 11,873, *p *< 0.0001).

**Table 4:** One hundred eighty-day follow-up cost comparison among medically ill patients

Follow-up cost	VTE		No VTE		*P*-value	Major bleeding		No major bleeding		*P*-value	Minor bleeding		No minor bleeding		*P*-value
	(n = 296)		(n = 10,839)			(n = 208)		(n = 10,927)			(n = 1465)		(n = 9670)		
	Mean	Std	Mean	Std		Mean	Std	Mean	Std		Mean	Std	Mean	Std	
**Unadjusted 180-day follow-up cost**	$52,248	$47,464	$24,038	$63,492	<0.0001	$33,533	$33,432	$24,621	$63,701	0.0003	$33,682	$38,061	$23,440	$66,165	<0.0001
**Adjusted 180-day follow-up cost**	$52,157	$24,389	$24,164	$11,418	<0.0001	$33,656	$18,196	$24,765	$11,974	<0.0001	$33,690	$14,398	$23,610	$11,873	<0.0001

Std, standard difference.

In the multivariate Cox proportional hazard regression model, two variables were significantly associated with a reduced risk of clinical VTE: thromboembolic stroke (hazard ratio [HR]: 0.42) and treatment with any anticoagulant (HR: 0.37). The median time to clinical VTE for patients receiving anticoagulant prophylaxis was 182 days, whereas for patients without anticoagulant prophylaxis it was only 27 days (*p *< 0.0001). When controlling for other factors, two significant independent predictors of major bleeding were found: more male patients than female patients had major bleeding during the follow-up period (HR: 1.42, *p *= 0.0162), and more patients in the western region of the United States than those in the north central region of the United States suffered major bleeding (HR: 0.30, *p *= 0.0493).

Different patterns were observed for patients with minor bleeding. Fewer patients admitted with chronic heart failure, thromboembolic stroke, or severe lung disease had minor bleeding relative to patients admitted with cancer (HR: 0.79, 0.64, 0.80, respectively) (Table 5).

**Table 5:** COX model for time to VTE, major bleeding, and minor bleeding events among medically ill patients

	Time to VTE		Time to major bleeding		Time to minor bleeding	
	Pr > Chi squared	Hazard ratio	Pr > Chi squared	Hazard ratio	Pr > Chi squared	Hazard ratio
Age, yr	0.9044	1.00	0.8416	1.00	0.1257	1.00
Male	0.4594	0.92	**0.0162**	**1.42**	0.1120	1.09
Northeast	0.4015	2.16	0.3896	0.55	0.4711	0.80
North Central	0.3526	1.75	**0.0493**	**0.30**	**0.0328**	**0.62**
South	0.3603	1.71	0.0583	0.32	0.0647	0.67
Elixhauser Comorbidity Index ≥2	0.3879	1.15	0.5041	0.86	0.8457	1.02
Prior VTE	0.0978	1.30	0.4936	1.28	0.5269	1.09
Chronic heart failure	0.3315	0.83	0.2288	0.74	**0.0068**	**0.79**
Thromboembolic stroke	**0.0017**	**0.42**	0.3261	1,25	**<0.0001**	**0.64**
Severe lung disease	0.8550	1.03	0.5226	0.87	**0.0020**	**0.80**
Acute infection	0.7596	1.05	0.2200	0.69	0.0910	0.87
Any anticoagulant	**<0.0001**	**0.37**	0.8602	1,03	0.5453	0.97

VTE, venous thromboembolism.

**Bold font **indicates statistical significance.

Table 6 presents the patient-day-based market share of anticoagulant treatment for patients who have diagnoses of acute medical illness. Among 11,135 patients, 3390 (30.44%) were treated with LMWH, 1629 (14.63%) with warfarin, 3531 (31.71%) with UFH, and 42 (0.38%) with fondaparinux within 180 days after index hospital discharge. The median duration of therapy was 4 days for LMWH and warfarin, 3 days for UFH, and 6 days for fondaparinux. Although warfarin was not administered to the majority of medically ill patients, the total days of warfarin use were the highest. Table 6 also demonstrates that the majority of prescriptions were administered in-hospital.

**Table 6:** Market share (patient-day based) of anticoagulants used in medically ill patients

Anticoagulant drug	No. of patients (%)	Days' supply per patient (mean)	Total days' supply	Days' supply per patient			No. of prescriptions	No. of prescriptions (inpatient)	No. of prescriptions (outpatient)
				*Q1*	*Median*	*Q3*			
LMWH	3390 (30.44)	6.30	21,367	2	4	7	17,358	16,865	493
Warfarin	1629 (14.63)	21.49	35,004	2	4	10	8207	7375	832
UFH	3531 (31.71)	5.24	18,508	1	3	6	18,189	15,162	3027
Fondaparinux	42 (0.38)	12.98	545	2	6	9	188	120	68

LMWH, low molecular weight heparin; UFH, unfractionated heparin.

## Discussion

Using a commercially available hospital drug database linked with outpatient claims, we evaluated the incidence of VTE, bleeding events, and associated costs in a cohort of medically ill patients with available prophylaxis information in both in-hospital and outpatient settings. This analysis extends the findings of previously published results in medically ill patients, which presented information limited to retail pharmacy-dispensed medications [13].

Recent research suggests that approximately two-thirds of medical inpatients with risk factors for VTE may receive some form of thromboprophylaxis and that, typically, thromboprophylaxis is maintained until hospital discharge, as is recommended by ACCP guidelines [4], in less than a quarter of these patients [5]. In a 2004 retrospective study by Goldhaber and colleagues, only 29% of hospital inpatients had received some form of thromboprophylaxis prior to their VTE events [6]. The International Medical Prevention Registry on Venous Thromboembolism (IMPROVE) found that only 6 in 10 eligible medically ill patients received prophylaxis [14], whereas the Epidemiologic International Day for the Evaluation of Patients at Risk for Venous Thromboembolism in the Acute Hospital Care Setting (ENDORSE) study found that 37% of patients with cancer or stroke received it [15]. One reason for this lack of adherence to guidelines may be that in the medically ill, the focus is necessarily on the patient's primary diagnosis, at the expense of VTE prophylaxis. For example, in patients with acute myocardial infarction (MI), ischemic cerebrovascular disease, or atrial fibrillation (AF), the prevention of arterial thrombosis is a primary goal and the prevention of VTE is secondary.

Results from the current analysis showed that despite existing guidelines, almost 47% of patients (n = 5203) did not receive appropriate anticoagulant prophylaxis, highlighting the underuse of appropriate anticoagulant prophylaxis in a wide range of medical inpatients at risk of VTE in the real world. Although clinical VTE rates during hospitalization were not evaluated in this analysis, the risk factors studied confirmed that appropriate anticoagulant prophylaxis use is significantly associated with an overall reduced risk of clinical VTE, including events occurring after hospital discharge.

This analysis also demonstrates that appropriate anticoagulant prophylaxis use, defined by recommendations in published guidelines [4], significantly contributes to a decrease in follow-up total healthcare costs. Indirect morbidity costs (lost income from work due to the condition or disability), and indirect mortality costs (lost income due to early mortality) associated with VTE, which we did not evaluate in this analysis, contribute to the overall cost of patient care. Therefore, improving rates of appropriate anticoagulant prophylaxis will reduce the burden of VTE on the US healthcare system.

There are limitations to our analysis. First, it is based on retrospective records, which cannot provide full information on how physicians decide whether to adopt the prophylaxis practices recommended in current guidelines. While a determination of appropriate anticoagulant use was based on the primary reason for hospital admission, it is possible that inappropriate anticoagulant prophylaxis may result from other factors or a secondary diagnosis. However, the hospital-linked database was validated and has been used for many retrospective studies.

The next set of limitations is typical of any retrospective claims database. While claims data are extremely valuable for treatment patterns, healthcare resource utilization, and costs, these data are collected for the purpose of payment rather than research. The presence of a diagnosis code on a medical claim is not necessarily proof of the presence of disease, as diagnoses may be incorrectly coded or included as rule-out criteria rather than actual disease. However, we applied detailed quality checks to the data set before starting the analysis in order to mitigate these problems. Over-the-counter medications (e.g. aspirin) and physician-provided samples were not measurable in the claims data. However, since our analysis is descriptive and based on evaluating the effect of anticoagulant prophylaxis on the incidence and costs of VTE in medically ill patients, we do not believe this lack of information on over-the-counter medicine would significantly affect our results.

This analysis suggests that in a large proportion of US patients diagnosed with an acute medical illness in the United States, the percentage of those who receive appropriate anticoagulant prophylaxis drugs is too low. An increase in this percentage would likely significantly decrease VTE rates and total follow-up healthcare costs. Among possible reasons for the underuse of appropriate prophylaxis may be a delay in adopting clinical practice guidelines by physicians, who often view guidelines as difficult to apply [15]. Physicians' attitudes, fears about increased risk of bleeding, and adherence problems resulting from limitations of current therapies might also contribute to this lack of prophylaxis use [14].

## Conclusions

Appropriate anticoagulant prophylaxis results in lower VTE event rates and total follow-up healthcare costs in medically ill patients. However, in real-world populations in the United States, adherence to guideline-recommended anticoagulant prophylaxis in patients diagnosed with an acute medical illness is low.

## Competing interests

Onur Baser and Li Wang declare no competing interests. Nishan Sengupta is a Johnson & Johnson employee and holds stock in Johnson & Johnson.

## Authors' contributions

All authors have made substantial contributions to the conception and design, analysis and interpretation of data, and drafting of the manuscript - and all have given final approval of the version to be published.

## Appendix

ICD-9-CM codes used to define medical conditions

1. **Chronic heart failure**: 398.9x, 402.01, 402.11, 402.91, 404.01, 404.03, 404.11, 404.13, 404.91, 404.93, 425.4x, 428.xx

2. **Thromboembolic stroke**: 433.xx, 434.xx, 435.xx, 436.xx

3. **Severe lung disease**: 466.xx, 480.xx, 481.xx, 482.xx, 483.xx, 484.xx, 485.xx, 486.xx, 487.xx, 490.xx, 491.xx, 492.xx, 493.xx, 494.xx, 495.xx, 496.xx, 500.xx, 501.xx, 502.xx, 503.xx, 504.xx, 510.xx, 511.xx, 512.xx, 513.0x, 514.xx, 515.xx, 516.xx, 518.0x, 518.2x, 518.3x, 518.4x, 518.5x, 518.81, 518.82, 518.83, 518.84, 518.89, 136.3

4. **Acute infection**: 003.1x, 008.xx, 020.2x, 022.3x, 027.xx, 036.2x, 038.xx, 041.xx, 054.5x, 098.89, 112.xx, 376.01, 478.21, 478.71, 528.3, 590.xx, 595.xx, 597.xx, 614.4, 681.xx, 682.xx, 790.7x

5. **Cancer**: 140.xx - 208.xx, 230.xx - 234.xx
